# Evolutionary and phylogenetic insights from the mitochondrial genomic analysis of *Diceraeus melacanthus* and *D. furcatus* (Hemiptera: Pentatomidae)

**DOI:** 10.1038/s41598-024-63584-w

**Published:** 2024-06-04

**Authors:** Lilian Cris Dallagnol, Fernando Luís Cônsoli

**Affiliations:** Insect Interactions Laboratory, Luiz de Queiroz College of Agriculture, University of São Paulo, Piracicaba, SP Brazil

**Keywords:** Mitochondria, Phylogeny, Sequence annotation, Evolutionary ecology, Molecular ecology, Molecular evolution, Phylogenetics, Mitochondrial genome, DNA sequencing, Next-generation sequencing

## Abstract

The mitochondrial genomes of *D. melacanthus* and *D. furcatus* were sequenced and used to investigate the phylogenetic relationships with 54 species of Pentatomidae. Their mitogenomes are 17,197 and 15,444 bp-long, respectively, including 13 protein-coding genes (PCGs), 2 ribosomal RNA genes, and 22/21 transfer RNA genes, with conserved gene arrangement. Leu, Lys, and Ser were the most common amino acids in their PCGs. PCGs evolutionary analysis indicated their mitogenomes are under purifying selection, and the most conserved genes are from the cytochrome complex, reinforcing their suitability as markers for molecular taxonomy. We identified 490 mtSSRs in 56 Pentatomidae species, with large variation and a positive correlation between mtSSR number and genome size. Three mtSSRs were identified in each *Diceraeus* species. Only the mtSSR in the *nad6* (*D. melacanthus*) and *nad4* (*D. furcatus*) appear to have application as molecular markers for species characterization. Phylogenetic analysis confirmed the monophyly of Pentatomidae. However, our analysis challenged the monophyly of Pentatominae and Podopinae. We also detected unexpected relationships among some tribes and genera, highlighting the complexity of the internal taxonomic structure of Pentatomidae. Both *Diceraeus* species were grouped in the same clade with the remaining Carpocorini analyzed.

## Introduction

The green-belly bugs *Diceraeus furcatus* and *D. melacanthus* (Hemiptera: Pentatomidae) are important pests of soybean, winter cereal, and especially maize in Brazil^1–4^. Their increased abundance is attributed to the agricultural system in practice in Brazil, with the continuous cultivation of maize following soybean harvest. This system provides year-round food and shelter for green-belly bugs and other pests^5–7^. Despite their relevance as agricultural pests, few molecular studies have been conducted on these species^8,9^, especially concerning their genetic delimitation, given their morphological similarity and considerable phenotypic plasticity^10^.

The identification of some insects is challenging and it requires additional tools, such as the use of integrative diagnostic methods based on molecular, morphological, and paleontological data^11^. In recent decades, the use of DNA barcode markers based on a short region of the mitochondrial cytochrome oxidase I gene (*cox1*) has been the most used molecular tool. The *cox1* became widely used for species recognition and delineation in various taxonomic groups^12^, although it has shown limited application for species delineation in taxonomic groups carrying low intraspecific variability^13^.

The mitochondrial genome (mtDNA) has emerged as a powerful tool for insect diversity and phylogenetic studies, expanding the potential pool of informative marker genes^13^. Its unique features, such as small size^14^, stable genetic composition^15^, maternal inheritance^16^, orthologous genes and low intermolecular recombination rates^17^, make it suitable for evolutionary studies. mtDNA serves as a source of molecular markers for population genetics, comparative evolution, divergence time analysis^18–20^, and gene rearrangements^21,22^. mtDNA markers are also instrumental in species recognition and delineation^23^, resolving conflicts of species complexes and in studying the biodiversity of taxonomic groups lacking experts for identification.

The mitochondrial genome of insects is circular, compact and up to 18 kb. It typically comprises 37 genes, including 13 protein-coding genes (PCGs), 2 ribosomal RNA genes (rRNA), and 22 transfer RNA genes (tRNA)^14,24,25^. The gene arrangement is often conserved^26^ and even small changes can provide valuable insights into phylogenetic relationships among organisms^27^. In addition, insect mitogenomes usually have a large non-coding region (A + T-rich region), containing essential regulatory elements for replication and transcription^28,29^.

Microsatellites—repetitive sequences of simple tandem repeats (SSRs)—have been extensively used as genetic markers^30,31^. SSRs are abundant and carry high mutation rates^32^. They are present in coding and non-coding regions of eukaryotic and prokaryotic genomes^33–37^. SSRs can occur in the nuclear^36,38^, cytoplasmic, and mitochondrial genomes^39,40^. Their location in the mitogenome determines their functional role in gene regulation, development and evolution^36,41^. Although evidence suggests non-random SSR distribution in genomes, especially in plants^42^, information on the identification and characterization of mitochondrial microsatellites (mtSSRs) and their roles in insects is scarce and poorly understood.

In this study, we sequenced and described the mitogenomes of *D. furcatus* and *D. melacanthus*. We analyzed the structure and nucleotide composition of their mitochondrial genomes and compared them with that of other Pentatomidae species, providing insights for the development of additional molecular markers. Additionally, we conducted phylogenetic analyses to determine the systematic placement of *D. furcatus* and *D. melacanthus* relative to other Pentatomidae species.

## Material and methods

### Sample collection and mtDNA extraction

Males and females from a laboratory-reared population of *D. melacanthus* maintained under controlled conditions were individualized shortly after emergence. At the end of the pre-reproductive period, males and females were paired to form individual couples. Females with the highest number of fertile eggs were kept with their progeny until reaching the 3rd instar, when nymphs were subjected to mitochondrial DNA (mtDNA) extraction. Specimens of *D. furcatus* were provided by Dr. Antonio Ricardo Panizzi (Embrapa Trigo, Passo Fundo/RS). The obtained specimens underwent mtDNA extraction using the commercial Mitochondrial DNA Isolation Kit (Abcam®), following the manufacturer’s instructions. The samples were stored at − 20 °C and submitted for sequencing at the Multiuser Center for Agricultural Biotechnology, USP/Esalq.

### Sequencing, assembly, and annotation of the mitochondrial genome

Libraries for sequencing the mtDNA of *D. melacanthus* were prepared using the Nextera XT DNA Library Prep system (Illumina), while those for *D. furcatus* were prepared using the TruSeq Nano DNA Library Prep system (Illumina), given the total amount of mtDNA obtained for each of the studied species. The mitochondrial genome sequencing of *D. melacanthus* was conducted on the Illumina MiSeq V3 platform (2 × 250 bp), while that of *D. furcatus* was performed on the NextSeq platform (2 × 100 bp).

The quality of raw reads was assessed using FastQC version 0.11.9^43^. Adapters and low-quality bases were removed using Trimmomatic^44^, applying the following parameters: TruSeq2-PE.fa:0:30:10:8:true SLIDINGWINDOW:4:24 LEADING:3 TRAILING:3 HEADCROP:3 MINLEN:25.

The de novo assembly of the mitogenomes was performed using the NovoPlasty algorithm^45^, using a partial sequence of the cytochrome oxidase I gene from *D. melacanthus* (SRX2888372) deposited in the National Center for Biotechnology Information (NCBI) database (https://www.ncbi.nlm.nih.gov/) as a seed. Annotation of the assembled mtDNA was conducted using the MITOS Web Server for mitochondrial annotation (http://mitos.bioinf.uni-leipzig.de/index.py)^46^. The same software was employed to determine gene, rRNA, and tRNA boundaries, as well as to predict RNA secondary structures. GenomeVX (http://wolfe.ucd.ie/GenomeVx/)^47^ was used to visualize the complete mitogenomes.

Nucleotide and amino acid composition, the relative synonymous codon usage (RSCU), the number of non-synonymous substitutions per non-synonymous site (*ka*), the number of synonymous substitutions per synonymous site (*ks*), and the ratio (*ka*/*ks*) for each protein-coding gene (PCG) were calculated in Mega X^48^. *Ka* and *Ks* values were obtained using trimmed PCG sequences. AT-GC content and skew statistics were calculated using CGView^49^.

The complete mitogenomes of *D. melacanthus* and *D. furcatus* were also utilized to identify short mitochondrial DNA sequence repeats (mtSSRs) with the MIcroSAtellite tool (https://webblast.ipk-gatersleben.de/misa/)^50^. The minimum repeat length used was ≥ 12 for mononucleotides, ≥ 6 for dinucleotides, ≥ 4 for trinucleotides, and ≥ 3 for tetra-, penta-, and hexanucleotides. The interruption between two microsatellites was considered as zero. To facilitate comparisons of genomes of different sizes, SSRs were standardized per 1 kilobase (kb) of genome, calculating the relative abundance (RA) and relative density (RD) for the identified mtSSRs. RA provides the total number of SSR classes per kb of genome, and RD offers the total length (in nucleotides) contributing to 1 kb of genome nucleotides^51^.

The mitochondrial genomes of *D. melacanthus* and *D. furcatus* have been deposited in GenBank under accession numbers PP235949 e PP235950.

### Phylogenetic analyses

Phylogenetic analyses were conducted using the mitogenomes of *D. melacanthus* and *D. furcatus*, and of 54 species of Pentatomidae available at the NCBI GenBank database (Table 1). Two additional mitogenomes from Aleyrodidae and Reduviidae were used as outgroups (Table 1). We pre-processed the dataset using the MitoPhAST V3.0 pipeline^52^. Nucleotide sequences of PCGs were extracted, translated into amino acids, and aligned using Clustal Omega^53^. Poorly aligned regions were removed with trimAI^54^, and a supermatrix was constructed with the concatenated alignments of PCGs in the following order: *nad1, nad4, nad3, cox3, cox1, nad4L, cytb, atp8, nad6, nad2, cox2, atp6*, and *nad5*. Phylogenetic analyses were performed using Maximum Likelihood (ML) and Bayesian Inference (BI) methods. ML analyses were conducted in IQ-TREE v1.6.10^55^, using the mtInv + F + R5 model, as determined by ModelFinder^56^ through the Akaike Information Criterion (AIC), with 100,000 bootstrap iterations. Bayesian analysis of site-homogeneous models was performed in MrBayes v. 3.2.7^57^. Two independent runs with four chains (three heated and one cold) were conducted simultaneously for 10 million generations, with sampling every 1000 generations and 25% of burn-in. The remaining samples were used to construct the consensus tree and Bayesian posterior probabilities. Another Bayesian analysis under a site-heterogeneous model was implemented using PhyloBayes MPI 1.5a on the CIPRES Science Gateway 3.3^58^. After removing constant sites from the alignment, two independent chains starting from a random tree were run under the CAT + GTR + C4 (GTR) and C6 models. The final trees were visualized using FigTree v.1.4.4 software (http://tree.bio.ed.ac.uk/software/figtree/). Tree topologies obtained with each phylogenetic analytical method used were compared using the IQ-TREE web server (http://iqtree.cibiv.univie.ac.at/)^59^. The data sets of P123 and AA were subjected to Kishino–Hasegawa (KH)^60^, Shimodaira–Hasegawa (SH)^61^, weighted Kishino–Hasegawa test (WKH)^60^, weighted SH test (WSH)^61^, expected Likelihood Weight (ELW)^62^, and the approximately unbiased (AU) tests^63^ with 1000 replicates.

**Table 1:** Mitogenomes of Pentatomidae species and of two external groups (Aleyrodidae and Reduviidae) used in the analyses to determine the phylogenetic relationships and systematic positioning of two *Diceraeus* species.

Species	Code	Family	Subfamily	Tribe	Access number	References
*Anaxilaus musgravei*	Anmu	Pentatomidae	Pentatominae	Antestiini	MW679031.1	^29^
*Arma chinensis*	Arch	Pentatomidae	Asopinae	–	NC058611	^64^
*Arma custos*	Arcu	Pentatomidae	Asopinae	–	NC051562.1	^65^
*Brachymna tenuis*	Brte	Pentatomidae	Pentatominae	Sephelini	NC042802	^66^
*Carbula sinica*	Casi	Pentatomidae	Pentatominae	Eysarcorini	NC037741	^67^
*Catacanthus incarnatus*	Cain	Pentatomidae	Pentatominae	Catacanthini	NC042804	^66^
*Caystrus obscurus*	Caob	Pentatomidae	Pentatominae	Caystrini	NC042805.1	^66^
*Cazira horvathi*	Caho	Pentatomidae	Asopinae	–	NC042817.1	^66^
*Dalpada cinctipes*	Daci	Pentatomidae	Pentatominae	Halyini	NC058967	^68^
*Dalsira scabrata*	Dasc	Pentatomidae	Phyllocephalinae	Phyllocephalini	NC037374	^67^
***Diceraeus furcatus***	Difu	Pentatomidae	Pentatominae	Carpocorini	PP235949	This study
***Diceraeus melacanthus***	Dime	Pentatomidae	Pentatominae	Carpocorini	PP235950	This study
*Dichelops melacanthus*	Dime	Pentatomidae	Pentatominae	Carpocorini	BK059216	^69^
*Dinorhynchus dybowskyi*	Didy	Pentatomidae	Asopinae	–	NC037724.1	^70^
*Dolycoris baccarum*	Doba	Pentatomidae	Pentatominae	Carpocorini	KC460537.1	^71^
*Eocanthecona furcellata*	Eofu	Pentatomidae	Asopinae	–	MZ440302.1	^72^
*Eocanthecona thomsoni*	Eoth	Pentatomidae	Asopinae	–	NC042816.1	^66^
*Erthesina fullo*	Erfu	Pentatomidae	Pentatominae	Halyini	MW206767.1	^73^
*Eurydema dominulus*	Eudo	Pentatomidae	Pentatominae	Strachiini	NC044762	^74^
*Eurydema gebleri*	Euge	Pentatomidae	Pentatominae	Strachiini	NC027489	^32^
*Eurydema liturifera*	Euli	Pentatomidae	Pentatominae	Strachiini	NC044763	^74^
*Eurydema maracandica*	Euma	Pentatomidae	Pentatominae	Strachiini	NC037042.1	^68^
*Eurydema oleracea*	Euol	Pentatomidae	Pentatominae	Strachiini	NC044764	^74^
*Eurydema qinlingensis*	Eoqi	Pentatomidae	Pentatominae	Strachiini	NC044765	^74^
*Eysarcoris aeneus*	Eyae	Pentatomidae	Pentatominae	Eysarcorini	MK841489	^75^
*Eysarcoris annamita*	Eyan	Pentatomidae	Pentatominae	Eysarcorini	MW852483	^76^
*Eysarcoris gibbosus*	Eygi	Pentatomidae	Pentatominae	Eysarcorini	MW846868	^76^
*Eysarcoris guttigerus*	Eygu	Pentatomidae	Pentatominae	Eysarcorini	NC047222	^77^
*Eysarcoris montivagus*	Eymo	Pentatomidae	Pentatominae	Eysarcorini	MW846867	^76^
*Eysarcoris rosaceus*	Eyro	Pentatomidae	Pentatominae	Eysarcorini	MT165687.1	^76^
*Eysarcoris ventralis*	Eyve	Pentatomidae	Pentatominae	Eysarcorini	MT165688	^76^
*Euschistus heros*	Euhe	Pentatomidae	Pentatominae	Carpocorini	BK059218	^69^
*Glaucias dorsalis*	Gldo	Pentatomidae	Pentatominae	Nezarini	NC058968	^68^
*Gonopsis affinis*	Goaf	Pentatomidae	Phyllocephalinae	Phyllocephalini	NC036745.1	^78^
*Graphosoma rubrolineatum*	Grru	Pentatomidae	Podopinae	Graphosomatini	NC033875.1	^79^
*Halyomorpha halys*	Haha	Pentatomidae	Pentatominae	Cappaeini	NC013272.1	^80^
*Hippotiscus dorsalis*	Hido	Pentatomidae	Pentatominae	Caystrini	NC058969	^68^
*Hoplistodera incisa*	Hoin	Pentatomidae	Pentatominae	Hoplistoderini	NC042799	^81^
*Menida violacea*	Mevi	Pentatomidae	Pentatominae	Menidini	MK617948.1	^82^
*Neojurtina typica*	Nety	Pentatomidae	Pentatominae	Pentatomini	NC058971	^68^
*Nezara viridula*	Nevi	Pentatomidae	Pentatominae	Nezarini	EF208087.1	^83^
*Palomena viridissima*	Pavi	Pentatomidae	Pentatominae	Nezarini	NC050166.1	^84^
*Pentatoma metallifera*	Peme	Pentatomidae	Pentatominae	Pentatomini	NC058972	^68^
*Pentatoma rufipes*	Peru	Pentatomidae	Pentatominae	Pentatomini	MT861131.1	^85^
*Pentatoma semiannulata*	Pese	Pentatomidae	Pentatominae	Pentatomini	NC053653	Unpublished
*Picromerus griseus*	Pigr	Pentatomidae	Asopinae	–	MF805778.1	^86^
*Picromerus lewisi*	Pile	Pentatomidae	Asopinae	–	NC058610	^64^
*Piezodorus guildinii*	Pigu	Pentatomidae	Pentatominae	Piezodorini	BK059215	^69^
*Placosternum urus*	Plur	Pentatomidae	Pentatominae	Pentatomini	NC042812.1	^81^
*Plautia crossota*	Plcr	Pentatomidae	Pentatominae	Antestiini	NC057080.1	^87^
*Plautia fimbriata*	Plfi	Pentatomidae	Pentatomidae	Antestiini	NC042813.1	^81^
*Plautia lushanica*	Pllu	Pentatomidae	Pentatominae	Antestiini	NC058973.1	^68^
*Rubiconia intermedia*	Ruin	Pentatomidae	Pentatominae	Carpocorini	KP207596	^29^
*Stiretrus anchorago*	Stan	Pentatomidae	Asopinae	–	BK059217	^69^
*Scotinophara lurida*	Sclu	Pentatomidae	Podopinae	Podopini	NC042815.1	^81^
*Zicrona caerulea*	Zica	Pentatomidae	Asopinae	–	NC058303.1	^88^
*Aleurodicus dugesii*	–	Aleyrodidae	Aleurodicinae	–	NC005939	^89^
*Triatoma dimidiata*	–	Reduviidae	Triatominae	Triatomini	NC002609	^90^

## Results and discussion

### Organization and characteristics of mitogenomes

**Figure 1:**
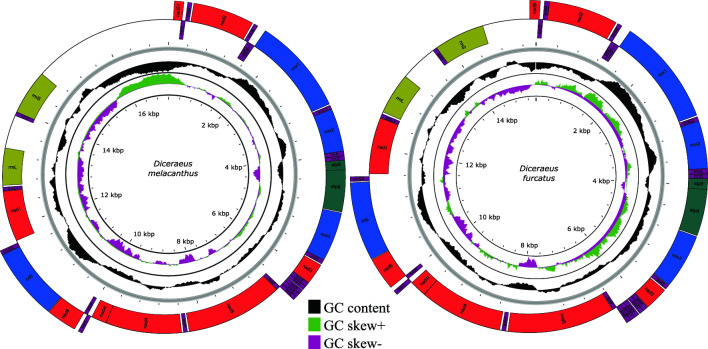
Circular maps of the mitochondrial genomes of (**a**) *Diceraeus melacanthus* (17,197 bp) and (**b**) *D. furcatus* (15,444 bp), illustrating the distribution of PCGs, tRNAs, rRNAs, the total GC content, and skews.

The mitochondrial genomes of *D. melacanthus* and *D. furcatus* are 17,197 and 15,444 bp—long circular, double-stranded DNA molecules, respectively. They contain 13 protein-coding genes (PCGs), 2 ribosomal RNA genes (rRNAs), and 22 (*D. melacanthus*) or 21 (*D. furcatus*) transfer RNA genes (tRNAs). The mitogenome of *D. furcatus* lacks the isoleucine tRNA gene (*trnI*). Thus, the mitogenome of *D. melacanthus* carries a total of 37 genes, while that of *D. furcatus* 36 (Table 2). The pseudogene *nad5-1* was identified in both mitogenomes. These mitogenomes have a conserved gene arrangement, with 23 genes located on the sense strand and 14 (13 in *D. furcatus*) on the antisense strand (Fig. 1). The intergenic spacers ranged from 2 to 1281 bp in *D. melacanthus*, and from 1 to 591 bp in *D. furcatus*. Gene overlaps in their mitogenomes varied between 1 and 11 bp (Fig. 1).

**Table 2:** Nucleotide composition of the mitogenomes of *Diceraeus melacanthus* and *D. furcatus*.

	Gene	Size (bp)	T(U)	C	A	G	AT (%)	GC (%)	GT (%)	AT skewness	GC skewness
*D. melacanthus*	Full genome	17,191	315	150	425	110	740	260	425	0.149	− 0.154
	PCGs	10,533	388	149	325	138	713	287	526	− 0.088	− 0.038
	tRNAs	1397	380	106	377	137	757	243	517	− 0.004	0.128
	rRNAs	1448	431	95	311	163	742	258	594	− 0.162	0.264
	*rrnL*	657	423	110	292	175	715	285	598	− 0.183	0.228
	*rrnS*	791	437	83	323	153	760	236	590	− 0.150	0.297
*D. furcatus*	Full genome	15,444	299	164	425	112	724	276	411	0.174	− 0.188
	PCGs	10,809	381	154	324	141	705	295	522	− 0.081	− 0.044
	tRNAs	1401	378	106	380	136	758	242	514	0.003	0.124
	rRNAs	1449	429	97	309	165	738	262	594	− 0.163	0.260
	*rrnL*	657	420	113	292	175	712	288	595	− 0.180	0.215
	*rrnS*	792	436	85	323	157	759	242	593	− 0.149	0.298

The A + T content (AT%) in the mitogenome of *D. melacanthus* is 74%, with a lower AT% in PCGs (71.3%) than in rRNAs (74.2%) and tRNAs (75.7%) (Table 2), resulting in a mitogenome with a positive AT skew (0.149) and negative GC skew (− 0.154). The PCGs exhibit a slightly negative AT skew due to the biased AT% in some of the *nad* genes (*nad1, nad4, nad4L, nad5*) (Table 2). The tRNAs and rRNAs also have a negative AT skew. The AT% of the mitogenome of *D. furcatus* was 72.4%, with PCGs carrying lower AT% (70.5%) than rRNAs (73.8%) and tRNAs (75.8%). The AT skews of tRNAs (0.003) and of the complete mitochondrial genome (0.174) of *D. furcatus* are positive, while those of PCGs (− 0.188) and rRNAs (− 0.163) are negative. The negative AT skew of PCGs is also likely due to the *nad* genes (Table 2).

The biased AT% of the whole mitogenomes, PCGs, rRNAs, and tRNAs genes of *Diceraeus* is similar to that of other Pentatomidae species^76,91^. Small variations in AT% among different genes have been attributed to DNA repair, selection, and mutations that occur during the evolutionary process^81,92^.

Mitochondrial genomes exhibit the characteristic strand asymmetry, usually reflected by AT and GC skews. In this study, AT (positive) and GC (negative) skews resemble the patterns normally reported for insects^29,91–93^. The length of concatenated PCGs of *D. melacanthus* (10,533 bp) was close to that of *D. furcatus* (10,351 bp), corresponding to approximately 61 and 67% of their mitogenomes, similarly to various Pentatomidae^14,29,70,82,94^. Nine PCGs (*nad2, cox1, cox2, atp8, atp6, cox3, nad3, nad6, cytb*) are encoded on the main strand and four on the complementary strand (*nad5, nad4, nad4L, nad1*) (Fig. 1). *nad5* is the longest PCG (1,677 bp for both species), while *nad4L* the shortest (264 bp for both species). ATD (ATA, ATT, ATG) and TTG are the most common initiation codons used in PCGs as reported for other Pentatomidae^64,79,94–96^.

**Figure 2:**
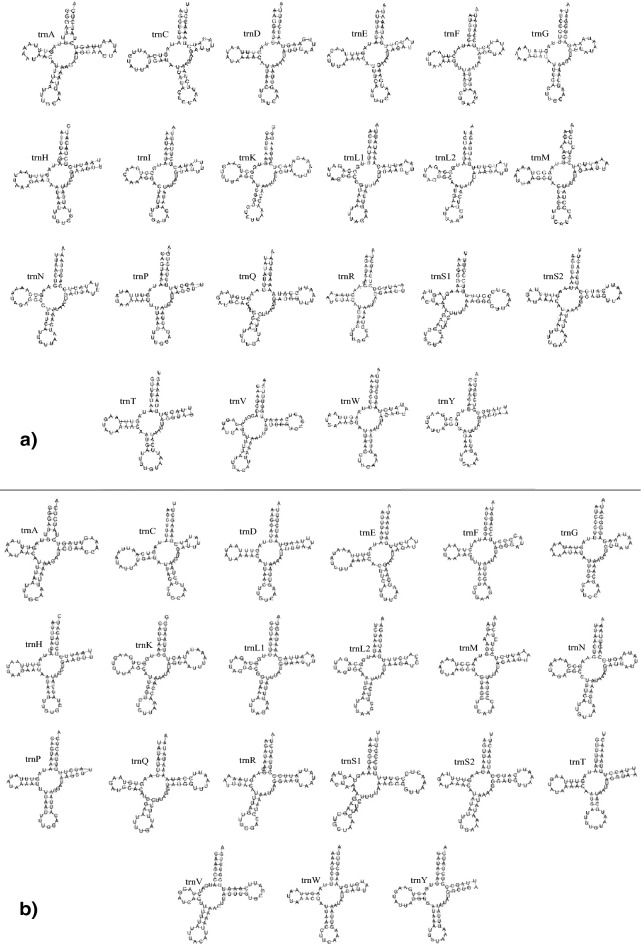
Secondary structure of tRNAs from **(a)** *D. melacanthus* and **(b)** *D. furcatus*. *trnA*—alanine; *trnC*—cysteine; *trnD*—aspartate; *trnE*—glutamate; *trnF*—phenylalanine; *trnG*—glycine; *trnH*—histidine; *trnI*—isoleucine; *trnK*—lysine; *trnL*—leucine; *trnM*—methionine; *trnN*—asparagine; *trnP*—proline; *trnQ*—glutamine; *trnR*—arginine; *trnS*—serine; *trnT*—threonine; *trnV*—valine; *trnW*—tryptophan; *trnY* – tyrosine.

Fourteen tRNA genes of *D. melacanthus* (t*rnM, trnW, trnL2, trnK, trnD, trnG, trnA, trnR, trnN, trnS1, trnE, trnT, trnS2*, and *trnI*) and 13 of *D. furcatus* (except *trnI*) are encoded on the main strand, with eight on the secondary strand (*trnQ, trnC, trnY, trnF, trnH, trnP, trnL1*, and *trnV*) (Fig. 1), all with the typical cloverleaf secondary structure (Fig. 2)^64,76,94,95,97^. The *D. melacanthus* mitogenome carries 20 tRNAs common to most metazoans, contrary to information from another mitogenome of the same species, which reports the absence of *trnI*^69^. In our study, the mitogenome of *D. furcatu*s presented 19 out of the 20 commonly reported tRNAs.

The loss of tRNA is considered rare in insect mitogenomes^98^, although many reports can be found. The gene cluster *trnT*, *trnQ*, and *trnM* was not found in the mitogenome of the hemipteran *Nabis apicalis*^99^; *trnI* was not detected in *Aleurochiton aceris*^89^; *trnS1* and *trnQ* were lost by *Aleurodicus dugesii*^89^; *Aleurocanthus camelliae* lacks *trnI*^100^; *Neomaskellia andropogonis* does not have *trnA*, *trnR*, trnN*, trnI*, and *trnS2*^89^. The loss of mitochondrial genes can be tolerated by the cell when their function becomes unnecessary or when it is taken over by a substitute gene with a similar role, or yet because it has been functionally transferred to the nucleus. The transference of gene functions from the mitogenome to the nuclear genome has many advantages^101–105^, including effects on species differentiation and the functional evolution of mitogenomes^106^. Genes encoding tRNAs in mitogenomes exhibit a mutation rate five times higher than nuclear genes, which undergo intense purifying selection^107^. However, there is no evidence that this occurred with the absent tRNA in this study. Instead, its function may have been replaced by nuclear tRNAs, given the frequent use of the isoleucine amino acid. *trnL* and *trnS* were duplicated in both mitogenomes. *trnS* copies were located in the same strand (sense), while *trnL* in different strands. Gene duplications also contribute to tRNA gene number variation among taxa^108^, and the loss of mitochondrial tRNAs in eukaryotes is not uncommon^101^.

The 16S and 12S rRNA genes are in the antisense strand of the mitogenomes of *D. melacanthus* and *D. furcatus* (Figs. 1 and 1S). The larger rRNA subunit (16S) is located between *trnL1* and a spacer region, and has identical sizes in both species (657 bp). The smaller rRNA subunit (12S) is located between the *trnV* gene and another spacer region, with of 791 bp in length for *D. melacanthus* and 792 bp for *D. furcatus*.

We demonstrated that the mitogenomes of *D. melacanthus* and *D. furcatus* share the same base composition pattern and conservation of content and gene arrangement observed after comparative analyses with other Pentatomidae mitogenomes^14,29,70,82,94^.

**Figure 3:**
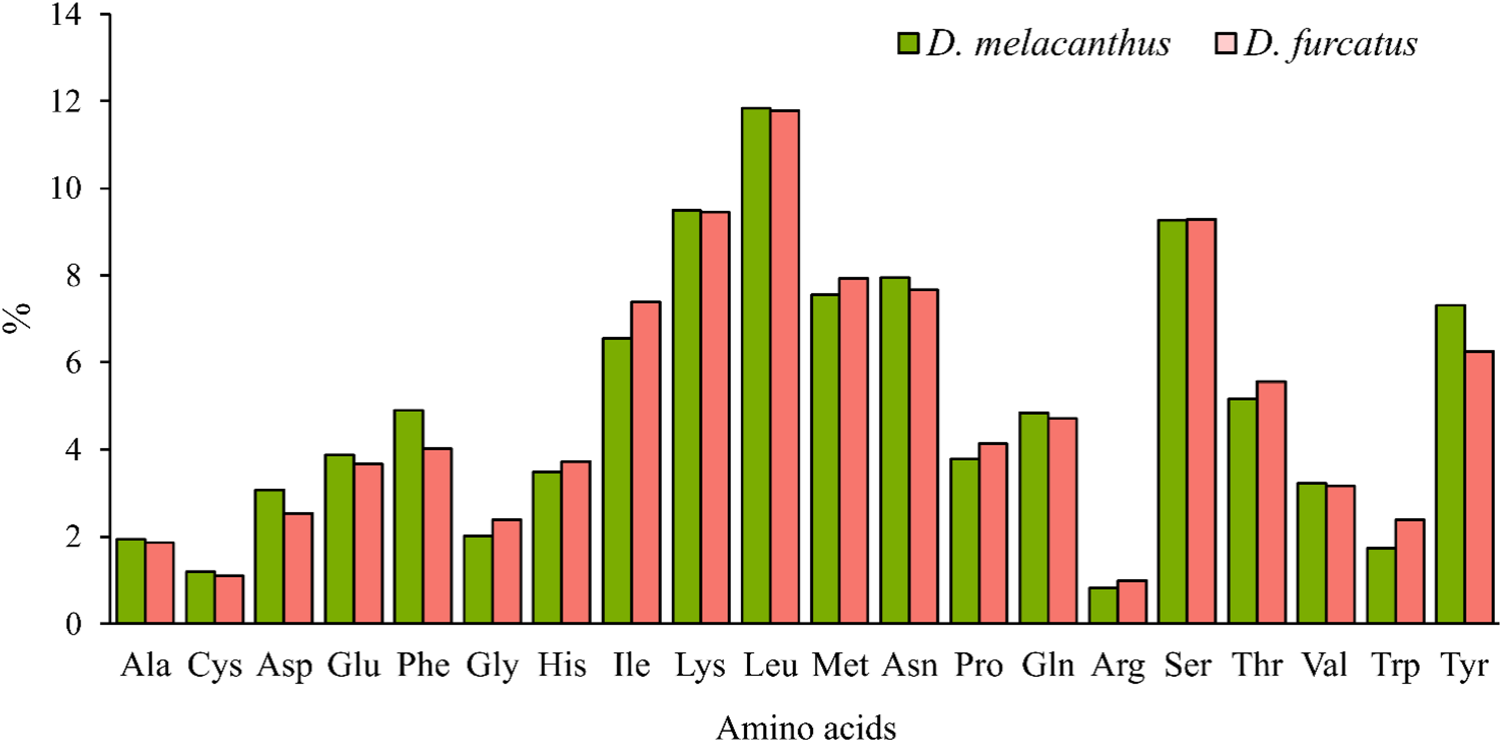
Amino acid composition of the protein-encoding genes of the mitogenomes of *Diceraeus melacanthus* and *D. furcatus*.

Leucine (Leu), lysine (Lys), serine (Ser), methionine (Met), and asparagine (Asp) are the most frequently used amino acids in the mitogenome of *Diceraeus* spp. (Fig. 3). Both *Diceraeus* species showed similar patterns of relative synonymous codon usage (RSCU). The most frequent codon (UUA–Leu2) used in *D. melacanthus* is the same of other Pentatomidae^91^, differing from the preferred AGA (Ser1) and UUA (Leu2) in the mitochondrial genome of *D. furcatus*. The preferred codons in PCGs showed a strong AT bias due to the predominance of A or T nucleotides in the third position of codons, following a pattern observed in Pentatomidae^69,91,94,109^.

**Figure 4:**
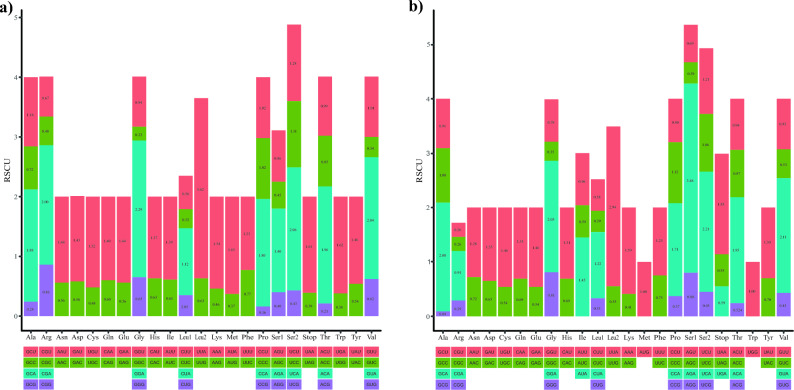
Relative synonymous codon usage (RSCU) of 20 amino acids and stop codons of the PCGs of the mitochondrial genome of (**a**) *Diceraeus melacanthus* and (**b**) *D. furcatus*.

There are 27 codons preferentially used (RSCU value > 1) for the 20 amino acids in PCGs in *D. melacanthus* and 26 in *D. furcatus* mitogenomes, representing less than half of the total set of codons (62 in *D. melacanthus*; 61 in *D. furcatus*) (Fig. 4). In *D. melacanthus*, all of them end in AT nucleotides, but only 84.6% in *D. furcatus*. Yet, in *D. furcatus* a large portion of synonymous codons are degenerate at the third nucleotide position, indicating greater selection pressure at this position from GC to AT^110^*.* The top five most used codons in *D. melacanthus* mitogenome are UUA (Leu2), GGA (Gly), UCA (Ser2), GUA (Val), and CGA (Arg); and AGA (Ser1), UUA (Leu2), UCA (Ser2), GUA (Val), GGA (Gly) in *D. furcatus*. Both *Diceraeus* species shared the preferential codon usage (Fig. 4).

**Figure 5:**
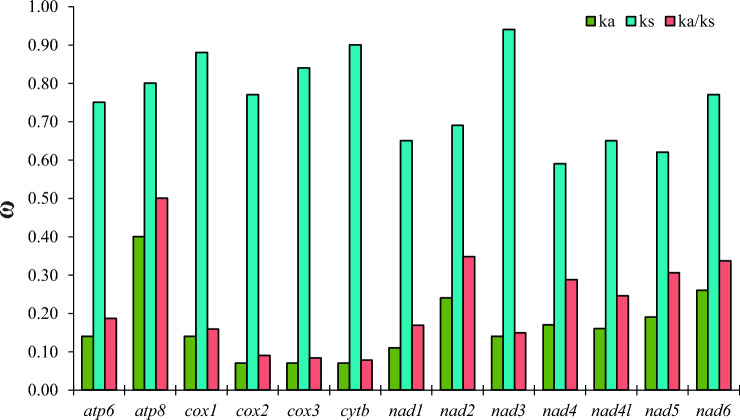
*ka* (non-synonymous substitutions), *ks* (synonymous substitutions) and *ka/ks* values for each PCG in the mitochondrial genomes of Pentatominae.

The evolutionary patterns of the subfamily Pentatominae were observed by calculating the synonymous substitution rate (*ks*), nonsynonymous substitution rate (*ka*), and the *ka*/*ks* ratio (ω), pairwise for each PCG (Fig. 5)^109,111^. All PCGs had ω < 1 (< 0.500), indicating evolution under purifying selection^29,109^. The most conserved genes with the lowest ω values are *cytb* (ω = 0.078), *cox3* (ω = 0.083), *cox2* (ω = 0.091), and *cox1* (ω = 0.159), are typically used as markers for molecular taxonomy, reinforcing their suitability for this purpose^12,29,95,109^. However, we were unable to efficiently amplify the barcode region of the *cox1* gene for *Diceraeus* species using the universal set of primers available (LCO1490/jgLCO1490 and HCO2198/jgHCO2198)^112,113^. Regarding the specific substitution rates of each gene, *Ks* ranged from 0.59 (*nad4*) to 0.94 (*nad3*), while *Ka* varied from 0.07 (*cox2, cox3, cytb*) to 0.40 (*atp8*).

### Mitochondrial microsatellites (mtSSRs)

The mitogenomes of *D. melacanthus and D. furcatus* presented 3 mtSSRs each, with two shared tetranucleotide mtSSRs equal in size (Table 3). The shared mtSSRs were located in the same coding gene (*nad4*) and intergenic region. The 18 bp-long trinucleotide of *D. melacanthus* was located in *nad6* gene, while the 12 bp-long tetranucleotide of *D. furcatus* was located in *nad5* gene (Table 3).

**Table 3:** Sequences of mtSSRs, number of repeats, total size, start and end positions in the mitogenome, and location region of mtSSRs found in *Dicereaus melacanthus* and *D. furcatus*.

Species	mtSSR	Nº repeats	Size (pb)	Start	End	Location region
*D. melacanthus*	AAAT	3	12	8419	8430	*nad4*
	TAT	6	18	10,168	10,185	*nad6*
	AATT	3	12	13,899	13,910	Intergenic region
*D. furcatus*	AAAC	3	12	6689	6700	*nad5*
	AAAT	3	12	8291	8302	*nad4*
	AATT	3	12	13,770	13,781	Intergenic region

**Figure 6:**
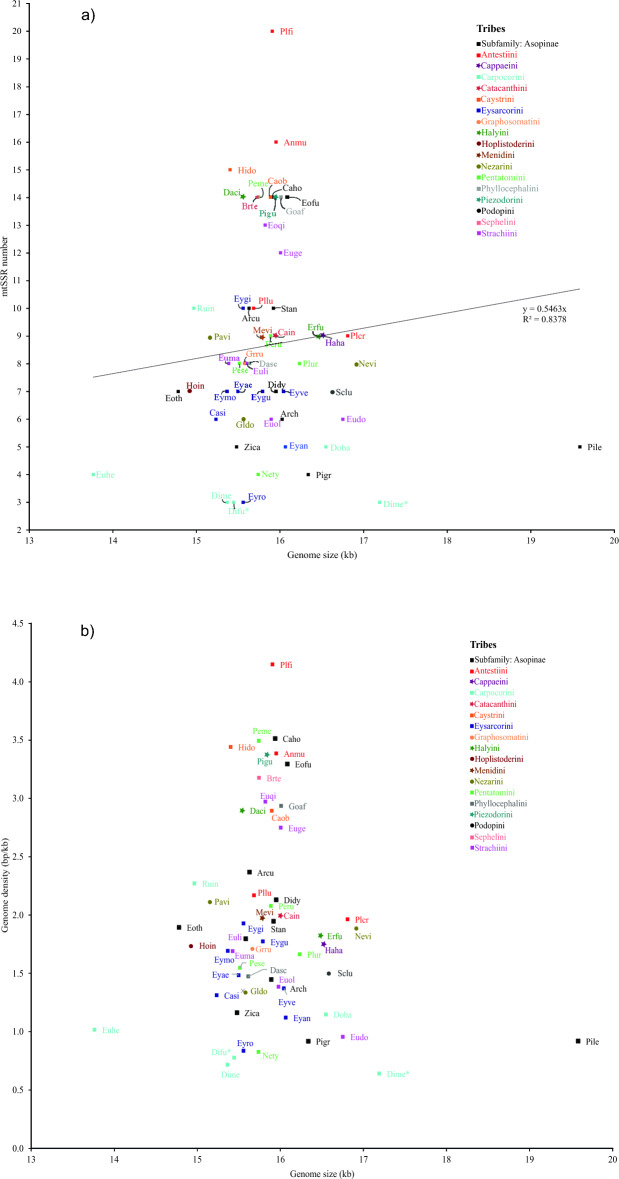
(**a**) Relationship between the number of mtSSRs and the mitogenome size (kb) from 56 Pentatomidae species; (**b**) Genome size (kb) and genomic density (bp/kb) of mtSSRs from 56 Pentatomidae species. The code names are based on the first two letters of the genus and species name (see Table 1).

The number of mtSSRs identified in *D. melacanthus* and *D. furcatus* was within the range observed in the mitogenome of the 56 pentatomid species analyzed (3–20 mtSSRs). A total of 490 mtSSRs were found, representing less than 1% of the mitochondrial genomes analyzed and within the range of microsatellites identified in the nuclear genome of insects (0.02–3.1%)^114^. The number of mtSSRs was positively related with the size of the mitochondrial genome (r^2^ = 0.83, *p* < 0.05), but with a wide variation in abundance among insect species (Fig. 6a), as reported elsewhere^39,51^. The lowest abundance (0.17 mtSSR/kb) and relative density (0.64 bp/kb) of mtSSRs were observed for *D. melacanthus*, while the highest values occurred in *Plautia fimbriata* (abundance = 1.26 mtSSR/kb; relative density = 4.15 bp/kb) (Fig. 6b). The abundance and relative density of mtSSRs in the mitogenome of pentatomids did not correlate with mitogenome size as previously reported in studies of insect genomes^114^. The GC content (%) of mtSSRs among species was very similar, ranging from 21.9 to 28.3%. The existence of a relationship between the number of microsatellites, abundance, and relative density with the size and GC content of mitochondrial genome in plants^51^ was not observed in pentatomids.

**Figure 7:**
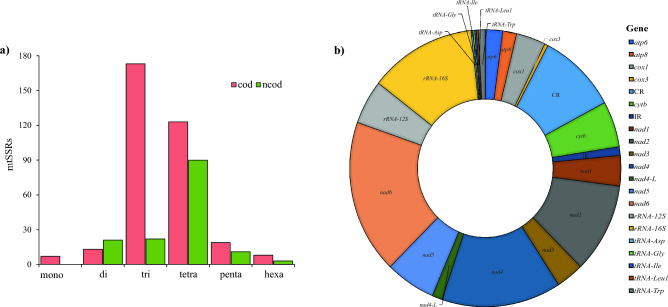
(**a**) Relationship between number of mtSSRs, size (bp) and location (coding or non-coding region) of 56 Pentatomidae mitogenomes cod = coding; ncod = non-coding; (**b**) Location of mtSSRs in the mitogenomes of 56 Pentatomidae species. CR: control region; IR: intergenic region.

The majority of mtSSRs of pentatomids were found in coding regions (70%), especially tri- and tetranucleotides (Fig. 7a). No mononucleotides were found in non-coding regions. Studies on mitogenomes of lower eukaryotes^115^ and animals^116^ also detected a higher abundance of mtSSRs in genic regions, possibly due to the prokaryotic origin of mitochondria (endosymbiont hypothesis)^116^. This is different from nuclear genomes in which SSRs are predominantly located in non-coding regions, including introns^117^. A significant portion of mtSSRs of pentatomids located in coding regions (Fig. 7b) is found in NADH dehydrogenase genes involved in the electron transport chain. *nad6* had the highest abundance of microsatellites (18%), followed by *nad4* (14%) and *nad2* (11%). Additionally, 13% of mtSSRs were located in *16S rRNA*.

**Figure 8:**
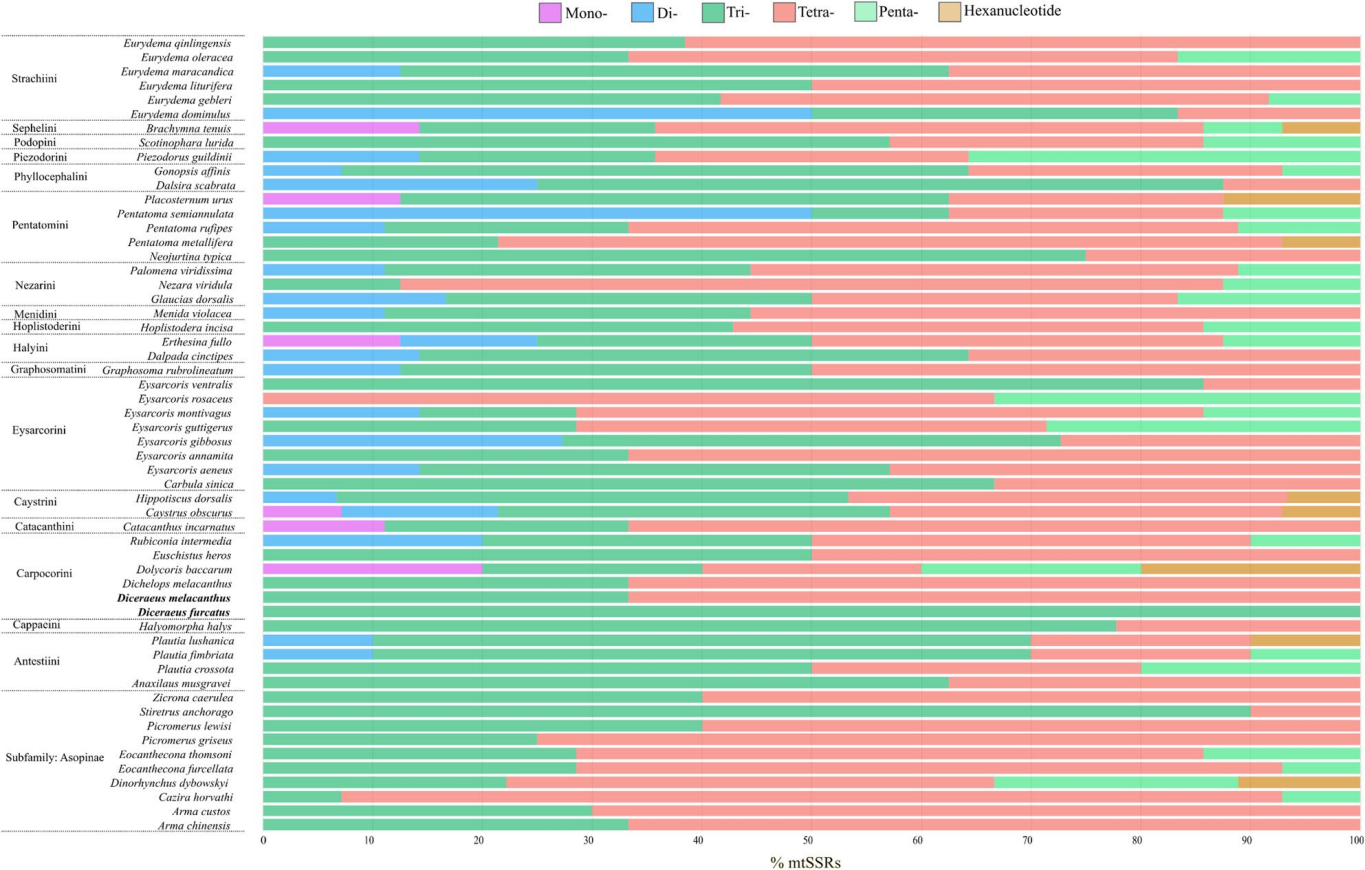
Relative percentage of six sizes of mtSSRs in 56 pentatomid mitogenomes. The percentages of mono-, di-, tri-, tetra-, penta-. and hexanucleotides are shown in different colors. The tribes of Pentatomidae are shown to the left of the species. Insects without a tribe belong to the subfamily Asopinae.

Tetranucleotides (43.1%) and trinucleotides (40.4%) were the most abundant types of mtSSRs, with all other types of mtSSRs representing less than 7% of abundance (Fig. 8). Studies on SSRs in insects are relatively uncommon but indicate a predominance of di- and trinucleotides^114^. In plants, where the majority of such studies are conducted, mono-, di-, and trinucleotides often predominate^39,118–122^. Among all mtSSRs in the mitogenomes of pentatomids, 98.8% of them had only A and T nucleotides in their composition. Dinucleotides were represented only by 6–9 repeats of AT/TA. Motifs TAT (27.1%), TAA (21%), and ATA (17.4%) with 4–6 repeats prevailed in trinucleotides. In tetranucleotides, 3–4 repeats of motifs AAAT (26.2%), TAAA (18.3%), and TTAT (14%) were the most common. Pentanucleotides were predominantly composed of motifs AAATA (30%), AAAAT (16.6%), AAATT (10.0%), and AATAA (10.0%), all with 3 repeats. Mono- and hexanucleotides represented only 1.4% and 1.6% of the total mtSSRs of pentatomids, respectively.

### Phylogenetic relationships in *Pentatomidae*

**Figure 9:**
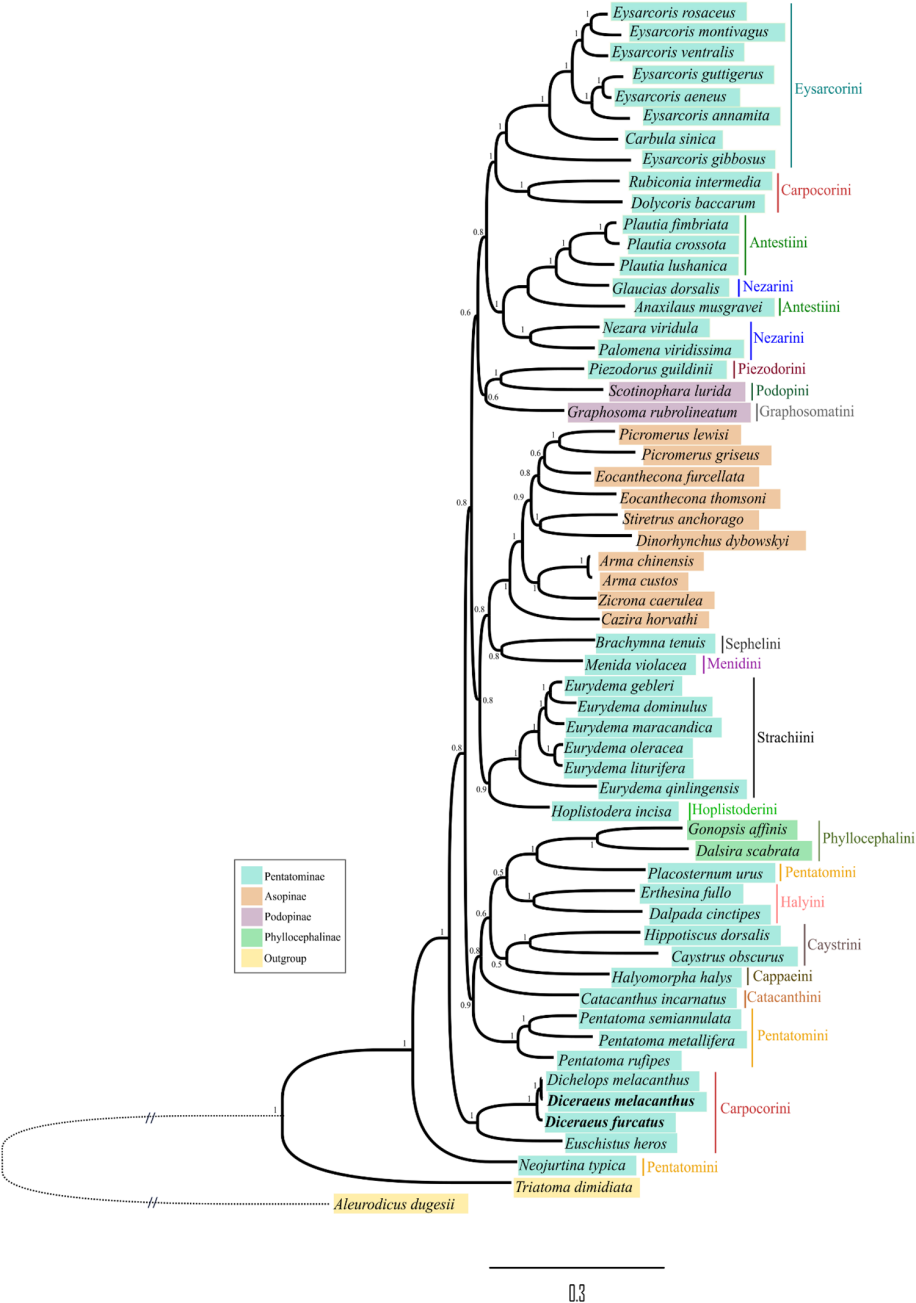
Phylogenetic relationships of tribes within Pentatomidae reconstructed from mtDNA sequences of 13 PCGs using the BI method. The numbers on the branches are the posterior probabilities (PP). The length of the branches is proportional to the genetic distance. Outgroup branches that don’t follow the scale are dashed. The subfamilies of Pentatomidae are shown by the colors in each species, and the tribes by vertical bars.

Four phylogenetic trees were obtained using different analytical methods (ML, CAT + GTR + C4, C60, and BI) of concatenated amino acid sequences of PCGs from 56 species of Pentatomidae (+ 2 outgroup species) (Fig. 9 and 2S). Topology tests (Table 1S) indicated tree topologies produced using CAT + GTR + C4, C60, and BI methods resulted in significant different log-likelihoods (*p* < 0.05). We chose to explore the BI tree due to the highest posterior probability value obtained (Fig. 9). The recovered phylogeny demonstrated that the Pentatomidae family is a monophyletic group with maximum posterior probability support (PP = 1), reinforcing previous reports^68,69,123–126^. However, our data challenge the monophyly hypothesis of Pentatominae and Podopinae, as representatives from both subfamilies intermingle, corroborating published findings^69^. Asopinae and Phyllocephalinae formed independent clades (PP = 1), although within Pentatominae. The monophyly and position of Asopinae within Pentatominae have been previously proposed based on morphology^124^ and molecular taxonomy^81,127^, highlighting the need for more detailed internal classification, especially because Asopinae is not divided into tribes^126,128,129^. *Neojurtina typica* (Pentatominae: Pentatomini) is placed as the oldest representative within the family^94,95^. Other Pentatominae species are also dispersed on the tree, such as *Placosternum urus* and *Piezodorus guildinii*, forming sister group relationships with representatives of Phyllocephalinae and Podopinae, respectively (Fig. 9).

The internal taxonomic structure of Pentatomidae has generally been considered stable. However, Pentatominae has been the subject of continuous questioning, revisions, and adjustments^125,128,129^. These questions arise because the studies that formed the basis for taxonomic classifications lacked high-quality phylogenetic methodology, had incomplete sampling, and poorly defined characters, limiting the impact of their findings^126^. Pentatominae comprises more than 40 tribes, including many genera that are challenging to accommodate in other subfamilies^129,130^.

The Pentatomini tribe is recognized as having less precise taxonomic definition^76^. The Nezarini and Antestini tribes are positioned within the same clade, suggesting a possible relationship due to their significant morphological similarity^126,131,132^. However, they are not considered monophyletic, although combined, they form a monophyletic group^94^.

Representatives of Halyini, also within Pentatominae, resolved into a single clade with high support probability, although positioned separately from most Pentatominae representatives (Fig. 9). The tribes Eysarcorini, Strachiini, Halyini, and Caystrini, and the genera *Pentatoma*, *Plautia*, and *Diceraeus* proved to be monophyletic. The two available mitochondrial genomes for *D. melacanthus* resolved into parallel branches forming a sister group with *D. furcatus*, in a clade with high posterior probability, which also includes another Carpocorini, *Euschistus heros*, in a slightly more external position. However, the Carpocorini tribe is not monophyletic, a result supported by recent analyses involving morphological and molecular data^126^.

*Eysarcoris gibbosus* was the first to branch off into the Eysarcorini clade, however, with *Carbula sinica* separating it from the other representatives of its genus. The positioning of *E. gibbosus* out of the *Eysarcoris* group supports the proposed reclassification of *E. gibbosus* to the genus *Stagnomus* (tribe Eysarcorini)^133^, which supports the closer systematically positioning of *E. gibbosus* to *C. sinica*^76,133,134^.

Members of the Piezodorini tribe have often been associated with members of the Menidini^131^, Nezarini^132^, Eurysaspini^129^, and the genus *Rhaphigaster*^126^ because of some common external morphological characteristics. However, molecular data based on mitogenome analysis did not support a relationship of Piezodorini with these groups but rather with the Podopinae subfamily (*Scotinophara lurida* and *Graphosoma rubrolineatum*), although with low support. The position of *Graphosoma* and *Scotinophora* raises some questions because they group with a clade of pentatomines, and positioned themselves as sister species. Others also agree with the monophyly of Podopinae, even though there are several apomorphies that disagree with this classification^126^.

Our data highlights the conservation of gene content and arrangement between *D. melacanthus* and *D. furcatus,* enhancing our understanding of the mitochondrial diversity in Pentatomidae. The identification and characterization of mtSSRs in coding regions offer new perspectives for phylogenetic and genetic studies within the family, suggesting potential molecular markers for population genetics and evolutionary analyses. The phylogenetic analysis underscores the need for revisions in internal taxonomic classification, challenging the monophyly of Pentatominae and Podopinae. In summary, this study not only contributes to the specific understanding of *D. melacanthus* and *D. furcatus* but also sheds light on broader issues of the taxonomy and evolution of Pentatomidae, providing insights for future research and refinements in taxonomic classifications.

### Supplementary Information

Supplementary Information.

## Data Availability

The new mitogenomes have been deposited in GenBank at https://www.ncbi.nlm.nih.gov/ under accession numbers PP235949 e PP235950. The other datasets analysed during the current study are available in GenBank, as referenced in the article.
