# Supplementary material for: Evolutionary and phylogenetic insights from the mitochondrial genomic analysis of Diceraeus melacanthus and D. furcatus (Hemiptera: Pentatomidae)
Source: Sci Rep. 2024 Jun 4;14:12861. doi: 10.1038/s41598-024-63584-w (PMC11150543; doi:10.1038/s41598-024-63584-w)
Supplement: Supplementary file 1 — Supplementary Information. [file 41598_2024_63584_MOESM1_ESM.docx]

**Supplementary Material**

**Evolutionary and phylogenetic insights from the mitochondrial genomic analysis of *Diceraeus melacanthus* and *D. furcatus* (Hemiptera: Pentatomidae)**

Lilian Cris Dallagnol; Fernando Luís Cônsoli*

University of São Paulo, Luiz de Queiroz College of Agriculture, Insect Interactions Laboratory, Piracicaba, SP, Brazil

Corresponding author: fconsoli@usp.br

ORCID

LCD: https://orcid.org/0000-0001-5604-0288

FLC: https://orcid.org/0000-0002-2287-0782

**
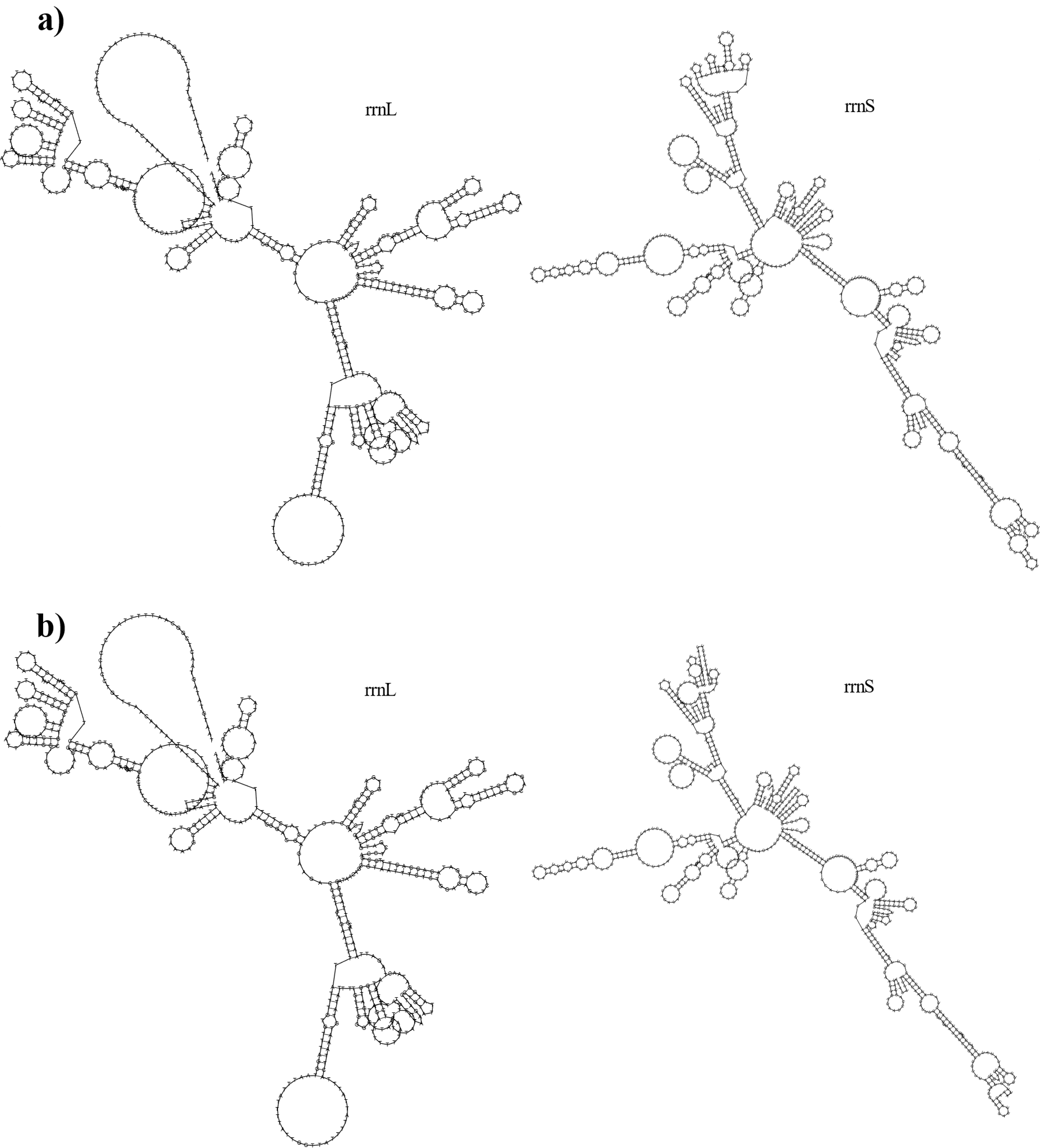
**

**Figure 1S** Secondary structure of large (*rrnL*) and small (*rrnaS*) ribosomal subunit of the mitochondrial genome of **(a)** *Diceraeus melacanthus* and **(b)** *D. furcatus*





**Figure 2S** Phylogenetic relationships of tribes within Pentatomidae reconstructed from mtDNA sequences of 13 PCGs using the CAT+GTR+C4, C60 and ML methods. The numbers on the branches are the posterior probabilities/bootstrap values. The length of the branches is proportional to the genetic distance. Branches that don't follow the scale are dashed. The subfamilies of Pentatomidae are shown by the colors in each species, and the tribes by vertical bars

**Table 1S** Topology tests of trees obtained using four different analytical methods for concatenated amino acid sequences from 56 species of Pentatomidae (+ 2 outgroup species)

| **Tree** | **logL** | **deltaL** | **p-KH** | **p-SH** | **p-WKH** | **p-WSH** | **c-ELW** | **p-AU** |
| --- | --- | --- | --- | --- | --- | --- | --- | --- |
| GTR | -65958.15 | 149.86 | 0.00* | 0.00* | 0.00* | 0.00* | 3.23e17* | 0.00* |
| C60 | -65885.34 | 77.05 | 0.01* | 0.01* | 0.01* | 0.02* | 0.00* | 0.01* |
| ML | -65808.29 | 0 | 0.99 | 1 | 0.98 | 0.99 | 0.98 | 0.99 |
| BI | -65887.04 | 78.75 | 0.01* | 0.02* | 0.01* | 0.03* | 0.01* | 0.01* |

deltaL: logL difference from the maximal logl in the set; *p*-KH: *p*-value of one-sided Kishino-Hasegawa test; *p*-SH*: p*-value of Shimodaira-Hasegawa test; *p*-WKH: *p*-value of weighted KH test; *p*-WSH: *p*-value of weighted SH test; c-ELW: Expected Likelihood Weight; *p*-AU: *p*-value of approximately unbiased (AU) test; * indicates statistical differences (*p*<0.05)
